# Systematic Analysis of Lysine Acetylation Reveals Diverse Functions in Azorhizobium caulinodans Strain ORS571

**DOI:** 10.1128/spectrum.03539-22

**Published:** 2022-12-08

**Authors:** Yanan Liu, Xiaolin Liu, Xiaoyan Dong, Zhiqiu Yin, Zhihong Xie, Yongming Luo

**Affiliations:** a CAS Key Laboratory of Coastal Environmental Processes and Ecological Remediation, Yantai Institute of Coastal Zone Research, Chinese Academy of Sciences, Yantai, China; b National Engineering Research Center for Efficient Utilization of Soil and Fertilizer Resources, College of Resources and Environment of Shandong Agricultural University, Taian, China; c University of Chinese Academy of Sciences, Beijing, China; d CAS Key Laboratory of Soil Environment and Pollution Remediation, Institute of Soil Science, Chinese Academy of Sciences, Nanjing, China; Connecticut Agricultural Experiment Station

**Keywords:** lysine acetylation, acetylome, *Azorhizobium caulinodans* ORS571, deacetylase, chemotaxis

## Abstract

Protein acetylation can quickly modify the physiology of bacteria to respond to changes in environmental or nutritional conditions, but little information on these modifications is available in rhizobia. In this study, we report the lysine acetylome of Azorhizobium caulinodans strain ORS571, a model rhizobium isolated from stem nodules of the tropical legume Sesbania rostrata that is capable of fixing nitrogen in the free-living state and during symbiosis. Antibody enrichment and liquid chromatography-tandem mass spectrometry (LC-MS/MS) analysis were used to characterize the acetylome. There are 2,302 acetylation sites from 982 proteins, accounting for 20.8% of the total proteins. Analysis of the acetylated motifs showed the preferences for the amino acid residues around acetylated lysines. The response regulator CheY1, previously characterized to be involved in chemotaxis in strain ORS571, was identified as an acetylated protein, and a mutation of the acetylated site of CheY1 significantly impaired the strain’s motility. In addition, a Zn^+^-dependent deacetylase (AZC_0414) was characterized, and the construction of a deletion mutant strain showed that it played a role in chemotaxis. Our study provides the first global analysis of lysine acetylation in ORS571, suggesting that acetylation plays a role in various physiological processes. In addition, we demonstrate its involvement in the chemotaxis process. The acetylome of ORS571 provides insights to investigate the regulation mechanism of rhizobial physiology.

**IMPORTANCE** Acetylation is an important modification that regulates protein function and has been found to regulate physiological processes in various bacteria. The physiology of rhizobium A. caulinodans ORS571 is regulated by multiple mechanisms both when free living and in symbiosis with the host; however, the regulatory role of acetylation is not yet known. Here, we took an acetylome-wide approach to identify acetylated proteins in A. caulinodans ORS571 and performed clustering analyses. Acetylation of chemotaxis proteins was preliminarily investigated, and the upstream acetylation-regulating enzyme involved in chemotaxis was characterized. These findings provide new insights to explore the physiological mechanisms of rhizobia.

## INTRODUCTION

Posttranslational modifications (PTMs) of proteins can affect their function and quickly change the physiology of bacteria to respond to changes in environmental or nutritional conditions (1, 2). Hundreds of PTMs have been identified, some of which have been extensively studied, such as acetylation, phosphorylation, and methylation (3). Acetylation refers to the transfer of acetyl groups to the N terminus of the protein (Nα-acetylation) or the ε-amino group on the side chain of lysine (Nε-acetylation) (4). Protein lysine acetylation and deacetylation was first discovered on the lysine of eukaryotic histones (5), and due to proteomics techniques, it was rapidly extended to nonhistone proteins, such as p53 and α-tubulin (6, 7).

To date, a large number of lysine-acetylated proteins have been identified in many eukaryotes, including human (8), mouse (9), Drosophila (10), yeast (11), and Arabidopsis (12). With the advancement of mass spectrometry-based proteomics methods, more than 50 acetylated protein data sets have also been obtained in prokaryotes, including Escherichia coli (13, 14), Salmonella enterica (15), Bacillus subtilis (16), and mycobacteria (17), in recent years (18). Protein acetylation is well conserved from bacteria to humans and is involved in important physiological processes like carbon/nitrogen metabolism, transcription, and the cell cycle (17, 19–21). Two types of enzymes playing a dynamic regulatory role in the acetylation process are the histone acetyltransferases (HATs) and histone deacetylases (HDACs). Bacterial genomes can encode between 1 and 70 HATs, each of which may have a different target, but they encode fewer HDACs (18). Protein acetyltransferases in prokaryotes are mostly homologs of yeast Gcn5 histone *N*-acetyltransferase (GNAT; PF00583) (22). Five HATs have been identified in E. coli, of which YfiQ (also known as Pat, PatZ, or Pka) has been extensively studied (15). In addition, the family of HDACs in organisms is divided into two main categories: NAD^+^-dependent silencing regulatory proteins (sirtuin family) and Zn^2+^-dependent deacetylases (Rpd3/Hda1 family) (23–26). Bacteria can encode predicted homologs of the corresponding classes, but only a few have been found to function as deacetylases so far (27). The sirtuin CobB is the only known global deacetylase in E. coli and S. enterica that can regulate a variety of physiological processes (28). Protein acetylation plays an important role in regulating bacterial metabolic pathways, as well as the flow of carbon metabolism. Multiple enzymes involved in central metabolic pathways can be acetylated, resulting in changes in activity (13, 19, 29–31). Acetylation of the chemotactic protein CheY was shown to inhibit its binding to motility-associated proteins, thereby affecting bacterial motility (32–34). In addition, Pat/CobB-mediated reversible acetylation of the transcription factor RcsB can regulate gene expression in E. coli (21). S. enterica serovar Typhimurium responds to different stresses in the host through reversible acetylation of PhoP in the two-component system, which is dynamically regulated by Pat/CobB (35). Another study demonstrates that acetylation affects the immunogenicity of the secretory proteins of Mycobacterium tuberculosis (17). Acetylation has been an important regulatory mechanism in diverse bacterial species studied thus far. But to our knowledge, large-scale identification of acetylated proteins has not been reported in rhizobia as yet.

Rhizobia fix atmospheric nitrogen through complex symbiotic relationships with legumes, which plays an important role in the sustainable development of agriculture (36). Azorhizobium caulinodans strain ORS571 has the dual capacity to fix nitrogen both as a free-living organism and in a symbiotic interaction with the leguminous plant Sesbania rostrata (37). Nodules formed by A. caulinodans ORS571 not only appear on the roots but also on the stems, which makes S. rostrata well fit as a pioneer plant for wetland improvement (38). It is necessary to fine-tune the physiological mechanisms of A. caulinodans ORS571 to respond to the complicated environment. Since the completion of genome sequencing (39), a number of genes involved in chemotaxis, nitrogen fixation, and nodulation have been characterized (40–42). Chemotaxis and motility enable soil bacteria to swim toward chemical attractors and avoid harsh environments (43), providing a competitive advantage for colonization of plant root surfaces (44). Chemotactic receptor IcpB, response regulator CheY, and chemotactic proteins CheA/CheZ in A. caulinodans ORS571 were characterized by our group (45–47). Both response regulators CheYs (CheY1 and CheY2) of A. caulinodans ORS571 played an important role in colonization of the host (40). The study confirmed that phosphorylation was involved in regulating the transduction of chemotactic signaling in A. caulinodans ORS571 (48), and therefore, acetylation of chemotactic proteins may become a new regulatory mechanism.

To further reveal the regulation of the physiological mechanism in A. caulinodans strain ORS571, we performed a systematic analysis of the lysine acetylome for the first time. A total of 2,302 acetylation sites in 982 acetylated proteins were identified in A. caulinodans ORS571. These acetylated proteins were subsequently clustered according to gene annotation, metabolic pathway, and protein domain. Acetylated proteins involved in important physiological processes like central metabolism, translation, chemotaxis, etc., were clustered to find key subnetworks. The construction of protein interaction networks lays the foundation for subsequent studies on the regulatory mechanisms of acetylation in specific physiological processes. We also investigated the effect of acetylation on the chemotactic protein CheY1 and found a new mechanism to regulate chemotaxis. In addition, the upstream deacetylase AZC_0414, which regulates acetylated proteins, was characterized for the first time. The role of AZC_0414 in chemotaxis was also analyzed. This study will provide a theoretical framework for the adaptation of ORS571 to the environment and the legume-rhizobium interactions.

## RESULTS AND DISCUSSION

### Identification of lysine acetylation in A. caulinodans ORS571.

**FIG 1:**
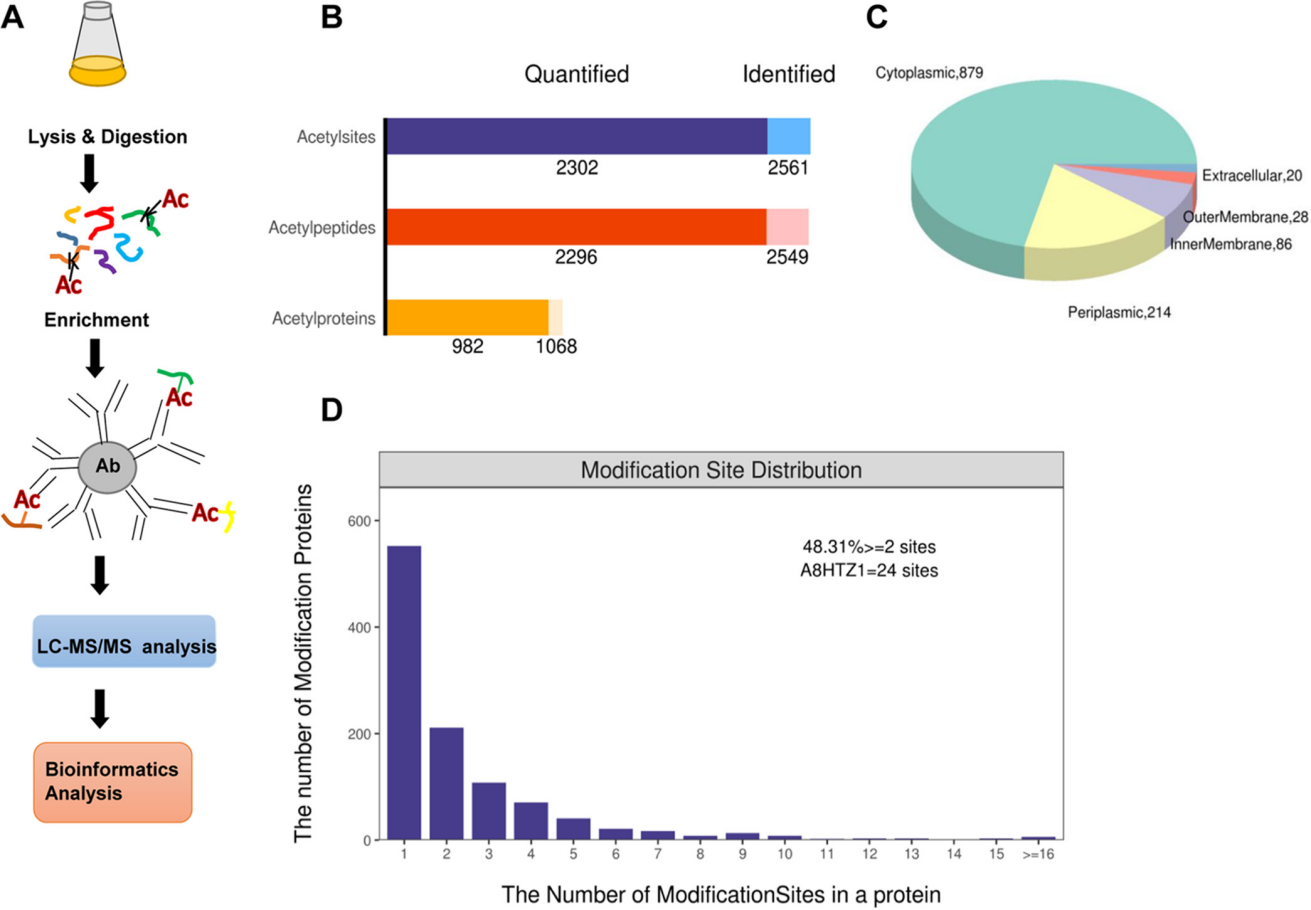
Characterization of lysine acetylation in A. caulinodans ORS571. (A) Schematic representation for global profiling of lysine acetylation in A. caulinodans ORS571. (B) Identification and quantitative analysis of acetylated proteins. Quantified acetylated peptides are acetylated peptides present in more than half of the biological replicates. (C) A pie chart representation of the distribution of the acetylated proteins identified according to subcellular location. (D) Distribution map based on the number of lysine acetylation sites.

The genome of A. caulinodans strain ORS571 was sequenced in 2008 (39), which provided a basis for us to study the lysine acetylation of the strain. A survey of the genome annotation revealed that A. caulinodans ORS571 encodes a large number of putative lysine acetyltransferases (Fig. S1 in the supplemental material), suggesting that acetylation may be widely distributed in this species. Previous studies in other systems have shown that acetylation begins to accumulate after bacterial growth enters the stationary phase (49, 50). Therefore, stationary-phase bacterial cells in minimal medium were used to determine the acetylome of A. caulinodans ORS571. An anti-acetyl lysine antibody was used to enrich acetyl lysine-containing peptides from trypsin digests. Enriched peptides were then analyzed by liquid chromatography-mass spectrometry (LC/MS) and identified by searching the corresponding database (Fig. 1A). The mass deviations of all identified acetyl peptides were mainly within 10 ppm, indicating that the quality accuracy of the MS data was reliable (Fig. S2A). Most of the peptides were between 7 and 20 amino acids in length, which is consistent with the digestive properties of trypsin (Fig. S2B). We identified a total of 2,302 acetylation sites on 982 acetylated proteins (Fig. 1B). These acetylated proteins contained 1 to 24 different lysine acetylation sites; approximately 48.3% of them contained two or more acetylation sites (Fig. 1D). Specifically, DNA-directed RNA polymerase subunit RpoC (A8HTZ1) had a staggering number of 24 acetylation sites. Details of the modification-specific peptides, identified lysine acetylation sites, and proteins are shown in Table S1. The investigation of the subcellular location showed that most acetylated proteins were distributed in the cytoplasm (879), and 214 proteins were located in the periplasmic space. One hundred fourteen proteins were located in the inner/outer membrane, and only 20 proteins were located in the extracellular region (Fig. 1C). Consistent with the subcellular localization of other organisms, most of the acetylated proteins were localized in the cytoplasm (11, 17, 51).

The above-described data constitute the first large-scale acetylome in rhizobia. Previous studies have revealed the proportions of acetylated proteins compared to total proteins in some prokaryotes to be 2.1% in Escherichia coli (14), 4.4% in Bacillus subtilis (16), 5.7% in Thermus thermophilus (52), 13.6% in Vibrio parahaemolyticus (53), 17.3% in Haloferax mediterranei (51), 20.5% in Streptococcus pneumoniae (54), 32.9% in Bacillus amyloliquefaciens DSM7 (55), and 45.7% in Deinococcus radiodurans (56). The proportion of acetylated protein to total protein in ORS571 was 20.8%, indicating that acetylation is an abundant posttranslational modification when compared to the extent of acetylation in other bacteria. Obtaining such abundant acetylome data also benefits from the improvement of technology (antibody specificity, recognition range, and sensitivity of mass spectrometry).

### Functional annotation and cluster analysis of acetylated proteins in *A. caulinodans* ORS571.

**FIG 2:**
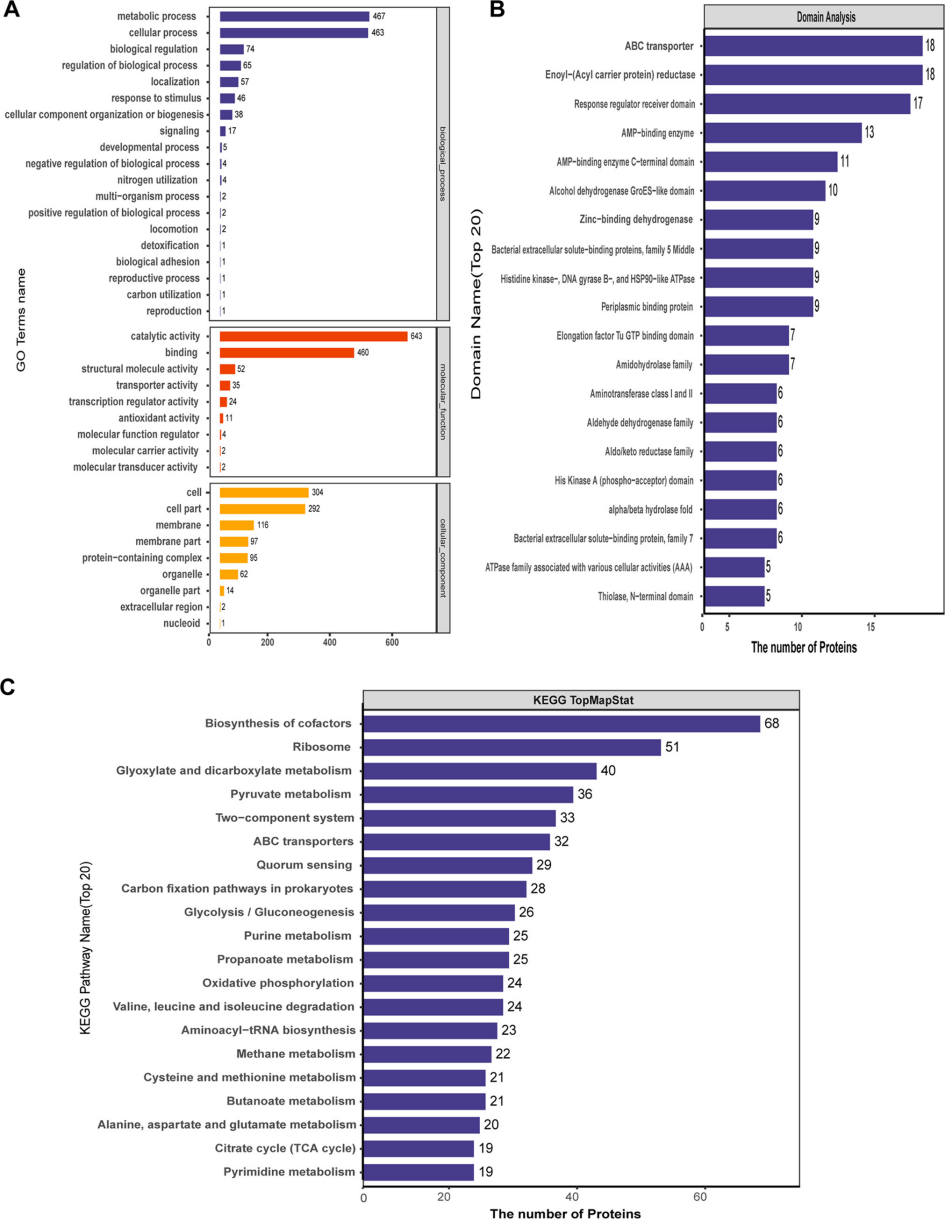
Analysis of lysine-acetylated proteins based on GO annotation (A), protein domains (B), and metabolic pathways (C) in A. caulinodans ORS571. (A) The vertical coordinate of the diagram shows that the functional annotation information contains biological process, molecular function, and cellular component, which are distinguished by different colors. (B) Protein domain classification analysis of all identified acetylated proteins. (C) KEGG metabolic pathway classification of the identified acetylated proteins.

To better understand the lysine acetylome in A. caulinodans ORS571, acetylated proteins were analyzed by using Gene Ontology (GO). As shown in Fig. 2A and Table S2, cluster analysis of the identified proteins according to their biological processes, molecular functions, and cellular compartments was performed. Cluster analysis for biological processes showed that most of the detected lysine-acetylated proteins were involved in metabolic processes (467 proteins) and cellular processes (463 proteins), consistent with findings in other bacteria (17, 57). In addition, acetylation was also involved in biological regulation (74 proteins), regulation of biological processes (65), localization (57), response to stimulus (46), cellular component organization or biogenesis (38), and signaling (17). In the GO molecular function category, the main functions of the identified acetylated proteins included catalytic activity (643), binding activity (460), structural molecular activity (52), transport (35), and transcriptional regulatory activity (24), etc. Catalytic and binding activities were the main functions performed by acetylated proteins, which is consistent with the study of Mycobacterium tuberculosis (17) and Haloferax mediterranei (51), indicating that the molecular functions of acetylated proteins are conserved from bacteria to archaea. After further cluster analysis of the distribution of acetylated proteins, it was found that acetylated proteins were mainly concentrated in the cell and cell part, which was consistent with the distribution of acetylated proteins. The GO analysis of acetylated proteins indicated that lysine acetylation was a widespread modification and might play important regulatory roles in biological processes and molecular functions in A. caulinodans ORS571.

Since domain prediction is of great significance for studying the key functional regions of proteins and their potential biological roles, an analysis of acetylated protein domains was performed. The top 20 enriched acetylated-protein domains are displayed in Fig. 2B and Table S3. There were 5 domains related to AMP/GTP binding, P loop, and ATPase domains, which play a role in nucleoside triphosphate hydrolysis. In addition, the enriched ABC transporter, enoyl-(acyl carrier protein) reductase, and response regulator receiver domains accounted for a significant proportion. The other enriched domains included alcohol dehydrogenase GroES-like domain, zinc-binding dehydrogenase, bacterial extracellular solute-binding proteins, periplasmic binding protein, amidohydrolase family, etc. Intriguingly, 6 acetylated proteins containing the histidine kinase A domain, which constitute a two-component signaling transduction system with their downstream target proteins, were enriched. These results indicated that a considerable number of proteins related to energy metabolism were acetylated, and many acetylated proteins related to transport and response regulation were found. Therefore, acetylation can be regarded as a common strategy for A. caulinodans ORS571 to regulate important physiological processes.

**FIG 3:**
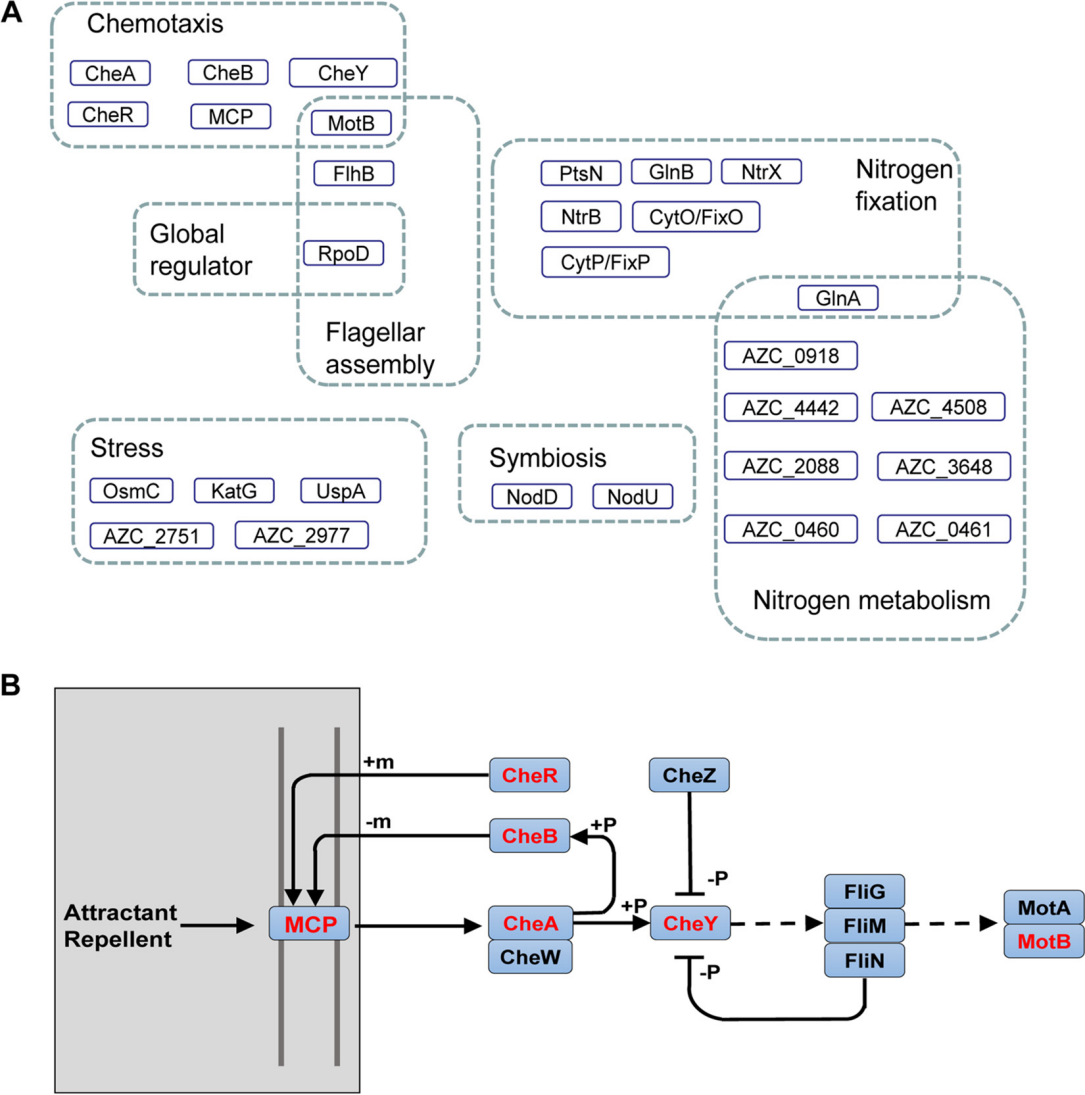
Acetylated proteins related to symbiosis with the host plant in A. caulinodans ORS571. (A) Global view of acetylated proteins involved in physiological processes like colonization, stress, symbiosis, and nitrogen fixation in A. caulinodans ORS571. (B) Acetylated proteins in the chemotaxis pathway of A. caulinodans ORS571 (acetylated proteins in red). “m” represents methylation, and “P” represents phosphorylation.

The acetylome data were mapped to the Kyoto Encyclopedia of Genes and Genomes (KEGG) pathway for annotation. As shown by the results in Fig. 2C and Table S4, the identified acetylated proteins (top 20) were mostly distributed in carbohydrate metabolism pathways, including glyoxylate and dicarboxylate, pyruvate metabolism, glycolysis/gluconeogenesis, propanoate metabolism, butanoate metabolism, and the citrate cycle (tricarboxylic acid [TCA] cycle). Proteins involved in energy metabolism, such as carbon fixation pathways, oxidative phosphorylation, and methane metabolism, as well as many proteins involved in the biosynthesis of cofactors and ribosomes, were also acetylated. As reported in other organisms, acetylated proteins were mainly involved in carbohydrate metabolism, ribosomes, and energy metabolism (54, 58), suggesting that important metabolic processes and protein synthesis are regulated by lysine acetylation in ORS571. Of particular interest is that acetylated proteins in ORS571 have also been identified in processes like chemotaxis, nitrogen fixation, symbiotic nodulation, stress, etc., playing an important role in the association with the host plant (Fig. 3A and Table S5). A number of acetylated proteins have been identified in the proteome analysis of the legume Medicago truncatula and its endosymbiont Sinorhizobium meliloti, but the analysis was mainly focused on the host plant rather than its rhizobia (36). Thus, the data obtained with A. caulinodans suggest that acetylation of symbiote-related proteins may be of importance. Several proteins of the chemotaxis pathway of A. caulinodans ORS571 were found to be acetylated. In this pathway, methyl-accepting chemotaxis proteins (MCPs) are methylated/demethylated by CheR/CheB, thereby changing bacterial sensitivity to external signaling stimuli (59). CheY and CheB can be regulated by CheA-mediated phosphorylation (60). The CheZ-mediated dephosphorylation mechanism plays an important role in the regulation of the motility of ORS571 (48). The identified acetylated chemotaxis proteins, including CheA, CheB, CheR, CheY1, and MCP (AZC_0821), are shown in Fig. 3B. This suggests that acetylation may play an important role in the chemotaxis and motility of ORS571 and implies that acetylation may be a new mechanism for regulating the chemotactic pathway of A. caulinodans ORS571, such as methylation and phosphorylation.

### Analysis of acetylated lysine motifs in *A. caulinodans* ORS571.

**FIG 4:**
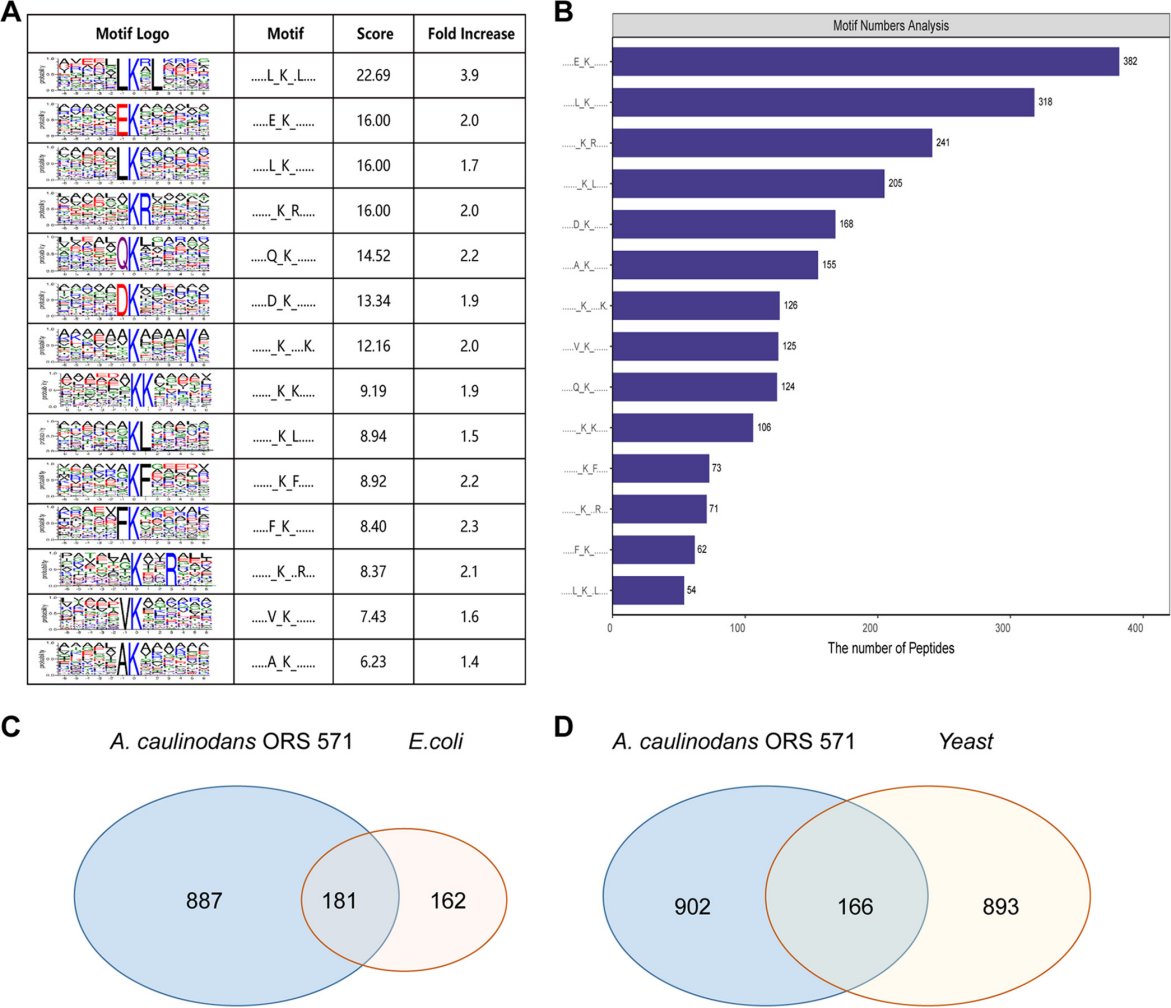
Evolutionary conservation analysis of acetylated proteins. (A) Acetylated lysine motifs analyzed by MEME software. The amino acid character size represents the frequency of occurrence of that amino acid residue in that position. The central K refers to the acetylated lysine. The motif score reflects the reliability of the predicted conserved motif, and a higher score means that the conserved motif is more specific and significant. (B) The number of identified peptides containing acetylated lysine in each motif. (C) Homologous acetylated proteins between A. caulinodans ORS571 and E. coli. (D) Homologous acetylated proteins between A. caulinodans ORS571 and yeast (S. cerevisiae).

It is generally necessary to recognize specific amino acid conserved motifs on the substrate protein through upstream enzymes (HATs and HDACs) in the process of protein modification. Therefore, it is important to investigate the conserved motifs of modified proteins for predicting the modification sites of protein substrate and substrate-enzyme interactions. The frequency distribution of amino acid occurrence was found by counting the number of occurrences of 6 amino acids upstream and downstream from the acetylation modification site on the modified peptide. This analysis revealed that 14 conserved motifs were identified in 2,296 acetylated peptides (Fig. 4A and Table S6). The most conserved motifs were LK_[Ac]_*L, EK_[Ac]_, LK_[Ac]_, and K_[Ac]_R, which were present in 54, 382, 318, and 241 acetylated peptides, respectively (K_[Ac]_ indicates the acetylated lysine, and * indicates a random amino acid residue) (Fig. 4B). The acetylated peptides possessing the above-named conserved motifs accounted for 43% of the total peptides identified, suggesting that they are the main characteristic motifs of acetylated protein substrates recognized by HATs. In addition, leucine and lysine at the +1 position, glutamine, and aspartate at the −1 position, and lysine at the +5 position also showed preferred status.

The conservation analysis of acetylated lysine motifs revealed that the preferred amino acid residues downstream from acetylated lysine were mainly alkaline residues, such as lysine and arginine. Previous studies have shown that the preferred alkaline residues were at the +3 to +6 positions in the Drosophila, human, and Candida albicans acetylomes (10, 61–63) and at the +1 to +3 positions in H. mediterranei (51). However, the preference for conserved motifs in bacteria varies from species to species. For example, histidine or tyrosine amino acid residues were preferred by E. coli at the +1 position, while glutamic acid, aspartic acid, lysine, or proline was preferred by B. subtilis (14, 16). In addition, glycine and glutamic acid were preferred at the −1 position in humans and Drosophila (10), but the preferred residues at the −1 position in ORS571 were leucine, glutamic acid, glutamine, and aspartate. A high abundance of aspartate and glutamic acid residues at the −1 position has also been observed in E. coli and T. thermophilus (1, 52). The differences in acetylated lysine motifs suggest that partially conserved protein motifs can be recognized by acetyltransferases in some organisms, and there may be novel acetyltransferases in ORS571 that recognize characteristic motifs.

To investigate the evolutionary conservation of acetylated proteins, we compared the data set with the acetylome of the model organisms E. coli and yeast (Saccharomyces cerevisiae) (11, 57). As shown in Fig. 4C and D, 181 of the 1,068 acetylated proteins found in A. caulinodans ORS571 were orthologs of acetylated proteins in E. coli and 166 were orthologs of acetylated proteins in yeast (Table S7). These results corroborate that lysine acetylation is a highly conserved posttranslational modification in all species, and we speculate that the identified acetylated protein plays an important role in A. caulinodans strain ORS571.

### Analysis of functional interaction networks of acetylated proteins in *A. caulinodans* ORS571.

**FIG 5:**
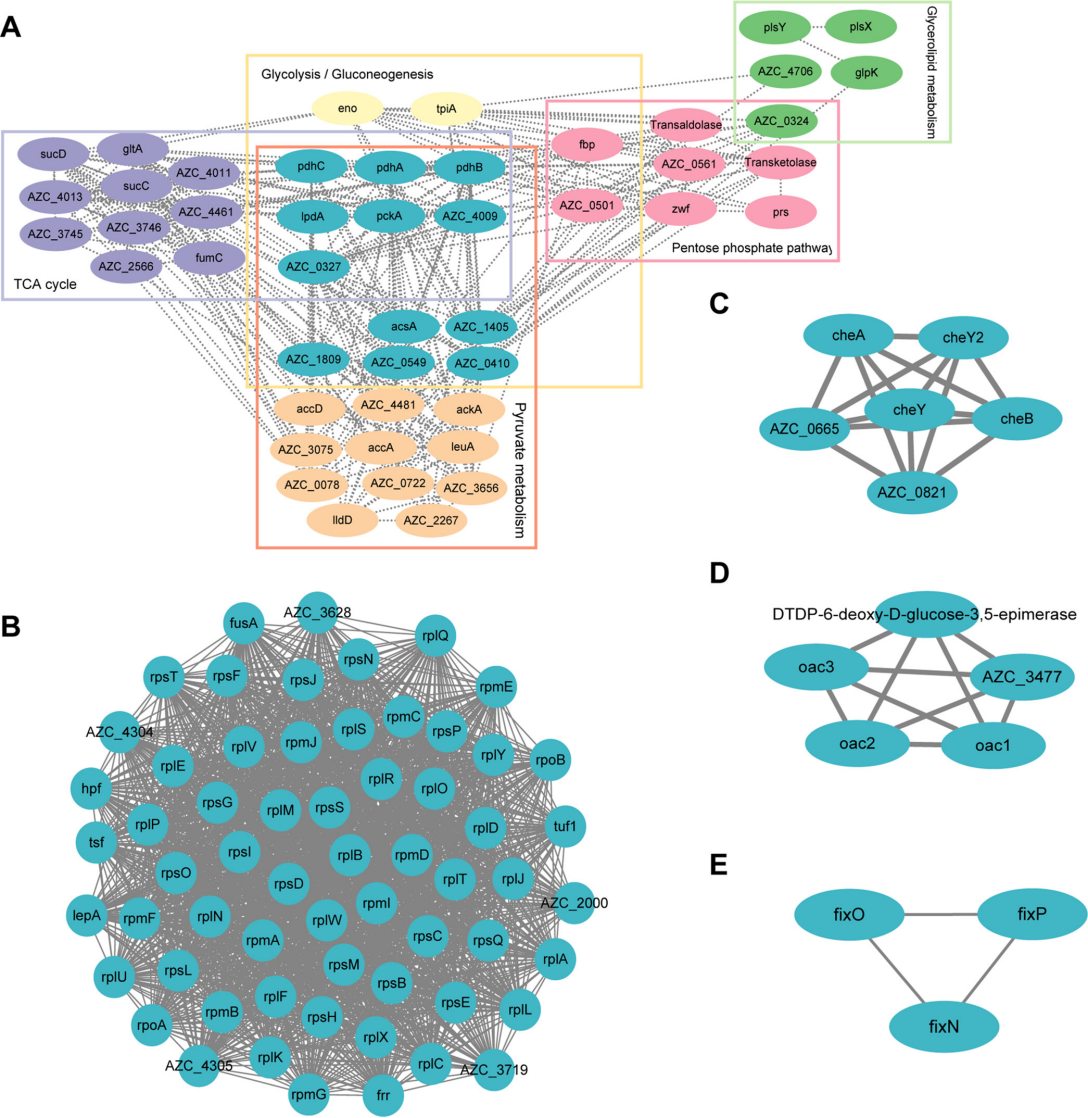
Protein interaction networks obtained with STRING (version 11.5) at confidence scores of ≥0.7 in the A. caulinodans ORS571 acetylome. (A) Subnetwork of enzymes mainly involved in the central metabolic pathway. (B) Subnetwork in ribosome and translational factors. (C to E) Subnetworks related to chemotaxis, extracellular polysaccharides, and nitrogen fixation.

In recent years, protein interaction networks have become an important means for in-depth exploration of omics data. They enable the visualization of key points in metabolic or signal transduction pathways of an organism to obtain a more comprehensive and systematic molecular-level model of cellular activity. An A. caulinodans ORS571 protein interaction network was constructed by mapping the 982 acetylated proteins in the data set to the STRING database. As shown in Fig. 5, five highly interconnected acetylated protein clusters were obtained by cluster analysis of the STRING database. Due to the large number of acetylated proteins of ORS571, the MCODE plugin was used to refine the clustering among the five protein clusters with high confidence (interaction score of ≥0.7). In cluster I, we found an interaction network containing a large number of central metabolic enzymes, including citrate cycle (TCA cycle), glycerolipid metabolism, glycolysis/gluconeogenesis, pyruvate metabolism, and pentose phosphate pathways (Fig. 5A). Consistent with previous studies, acetylation is present in most enzymes of central metabolic pathways in a variety of organisms (11, 15, 16, 64). In addition, numerous ribosome-related acetylated proteins were found to form a highly interconnected network (cluster II) (Fig. 5B). These highly interconnected acetylated ribosome-associated proteins are components of the ribosomal protein complex and play important roles during transcription (Table S8). Three other interesting subnetworks were also found in the study, namely, chemotaxis-related proteins, exopolysaccharide-related proteins, and nitrogen fixation-related proteins (Fig. 5C to E and Table S8). These results suggest that acetylated proteins are involved in evolutionarily conserved central metabolic pathways and protein translation processes and also play a role in some newly discovered biological processes. These data help us to further explore the physiological role played by lysine-acetylated proteins in ORS571.

### Acetylation analysis and verification of response regulator CheY1.

**FIG 6:**
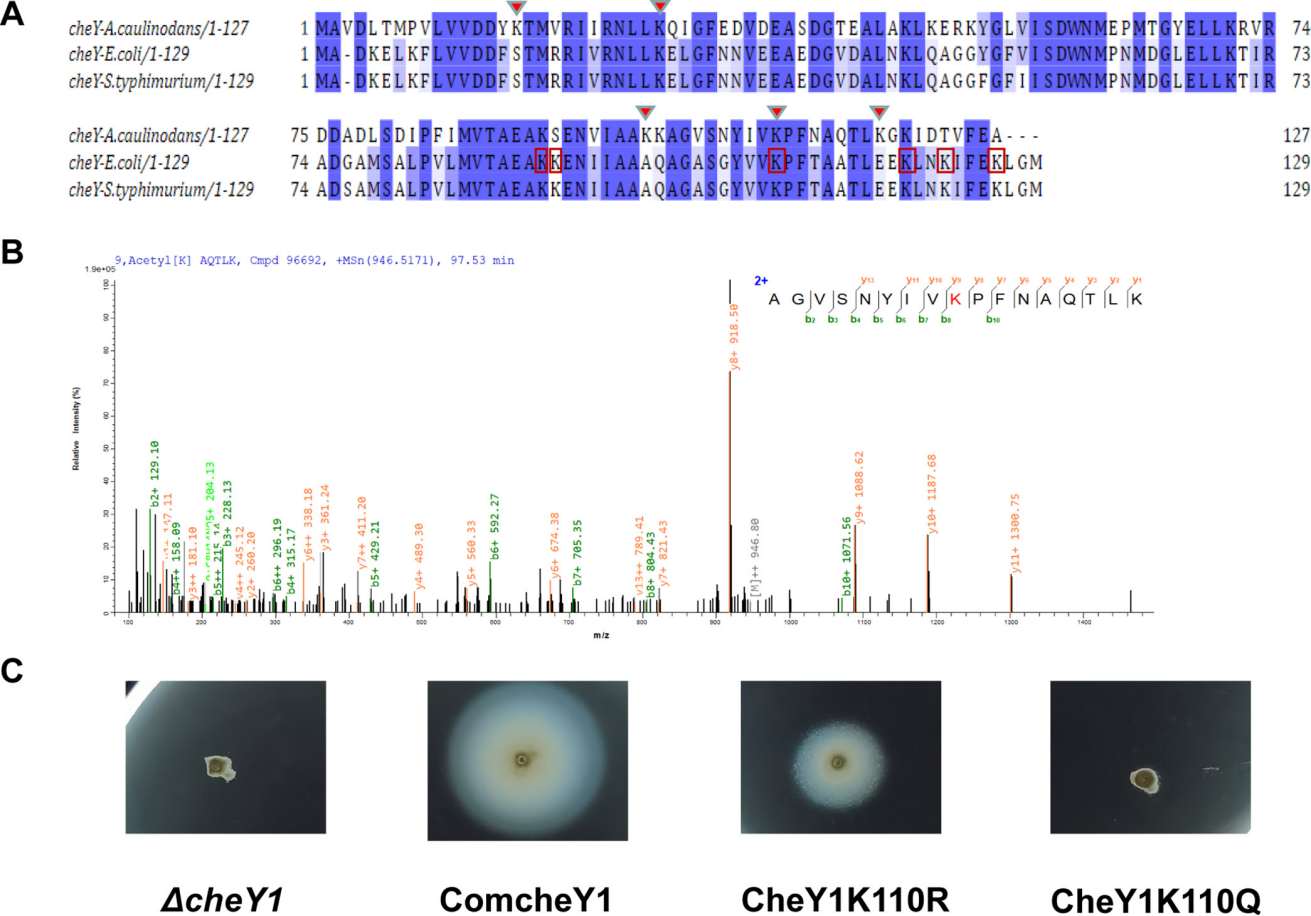
Acetylation of the chemotaxis protein CheY1 at the K110 site affects chemotaxis motility in A. caulinodans ORS571. (A) Sequence alignment of CheY1 in A. caulinodans ORS571 with those in E. coli and *S.* Typhimurium by Multiple Alignment using Fast Fourier Transform (MAFFT). The backgrounds of conserved residues are marked with blue and light blue according to the degree of conservation. Red triangles show the identified acetylation sites of CheY1 in A. caulinodans ORS571, and red boxes show the known acetylation sites of E. coli CheY. (B) MS/MS spectra of acetylated peptide AGVSNYIVK(Ac)PFNAQTLK of CheY1. (C) Wild-type or acetylated site-mutated fragments were cloned into the broad-host-range plasmid pBBR1MCS-2 and subsequently transformed into the *ΔcheY1* strain. On semisolid chemotaxis plates (0.3% agar), the K110R mutant restored part of the chemotactic motility, while the swimming motility of the K110Q mutant was lost.

Bacterial chemotaxis refers to the behavior of bacteria in response to environmental changes by regulating motility, which is essential for rhizobia to colonize the host. The response regulator protein CheY, a core chemotaxis protein, has an important role in bacterial motility. In A. caulinodans strain ORS571, which carries two *cheY* genes, encoding CheY1 and CheY2, CheY1 is primarily responsible for chemotaxis (48). Five acetylation sites (K16, K27, K100, K110, and K118) of CheY1 (AZC_0620) were identified in the above-described acetylome data of ORS571. Sequence alignment of ORS571 CheY1 with the CheY proteins of E. coli and *S.* Typhimurium showed that the three proteins displayed high similarity; in particular, the lysine residues K109 of E. coli and K110 of A. caulinodans ORS571 were conserved. (Fig. 6A). The MS spectra showed the conserved acetylation site (K110) of CheY1 in A. caulinodans ORS571 (Fig. 6B).

To investigate the effect of acetylation on the function of CheY1, several mutants were constructed using the primers listed in Table 1. The acetylation site K110 of CheY1 was replaced with arginine (R [K110R]) and glutamine (Q [K110Q]) to mimic constitutive deacetylation and acetylation, respectively (65). The wild-type fragment and point mutant fragment were cloned into the broad-host-range plasmid pBBRMCS-2, and the knockout mutant of CheY1 (*ΔcheY1*) and the complemented strain (ComcheY1) were used as controls. As shown by the images in Fig. 6C, the swimming motility of the *ΔcheY1* mutant was completely impaired, while the chemotactic behavior of ComcheY1 was restored. Like the *ΔcheY1* mutant, the swimming motility of the K110Q mutant was also lost. However, the K110R mutation restored part of the chemotactic motility, but it was still lower than that of the wild type. This indicated that deacetylation on the K110 site of CheY1 (K110R) inhibited its function but rescued the chemotactic motility to some extent, whereas acetylation of K110 (K110Q) destroyed the chemotactic motility function exerted by CheY1. Previous studies have confirmed that acetylation of CheY1 affected the binding to FliM, which led to reduced chemotaxis in E. coli (33). Therefore, we speculate that the acetylation of the CheY1 at the K110 site in ORS571 may regulate the binding to flagellar-complex proteins and thus affect chemotaxis. It was shown that phosphorylation of CheY in E. coli is involved in the regulation of chemotaxis (66, 67). CheY acetylation was subsequently demonstrated to regulate bacterial chemotaxis and to alter the mode of bacterial motility in coupling with phosphorylation (68–71). The phosphorylation and acetylation of CheY in E. coli results in different types and times to bind to flagellar motor proteins, and the two cooperatively regulate flagellar rotation, conferring flexibility to bacterial chemotaxis (68). These results suggest that protein acetylation modification is essential for the chemotactic motility in A. caulinodans ORS571 and that more acetylation-mediated regulatory mechanisms of chemotaxis deserve to be investigated.

**TABLE 1:** Primers, plasmids, and strains used in this study

Primer, plasmid, or strain	Sequence (5′–3′) or description	Purpose, reference(s), or source
Primers		
Com0620-EcoRI-up	CGGAATTCcacaaggcgttccggtcacg	Complemented strain (ComcheY)
Com0620-BamHI-dn	CGGGATCCtcaggcctcgaacacggtgtc	
Com0620-EcoRI-up	CGGAATTCcacaaggcgttccggtcacg	Mutation of K110R
K110R-1-DN	cccttcagcgtctgcgcattgaacggcctcacgatatagttgctcacgcc	
ComK110R-BamHI-dn	CGGGATCCtcaggcctcgaacacggtgtcgatcttgcccttcagcgtctgcg	
Com0620-EcoRI-up	CGGAATTCcacaaggcgttccggtcacg	Mutation of K110Q
K110Q-1-DN	cttcagcgtctgcgcattgaacggctgcacgatatagttgctcacgccgg	
ComK110Q-BamHI-dn	CGGGATCCtcaggcctcgaacacggtgtcgatcttgcccttcagcgtctgcgcattg	
18Δ0414UP-BamHI-F	CGGGATCCCGGATCGGATCATCCGAGGA	Construction of *ΔAZC_0414*
18Δ0414UP-R	ACATCCATCAGCTTCCTCCTGCTCGAAGATGCG	
18Δ0414DN-F	TCTTCGAGCAGGAGGAAGCTGATGGATGTGGCC	
18Δ0414DN-HindIII-R	CCCAAGCTTGAAGTGAGGGGATCGTGATTTC	
Δ0414-UP	GATGATGGCCAGCATCAGCG	Knockout validation
Δ0414-P6-DN	GGCAACGTGATCCAAACACGC	
Δ0414-P5-UP	GACCGCTCTCCTAGCAGATC	
Δ0414-DN	GCGAGATCGTCAGCTACGAAC	
Plasmids		
pBBR1MCS-2	Broad-host-range plasmid; Km^r^	87
pBBRCheY	pBBR1MCS-2 with *cheY* open reading frame and upstream promoter region; Km^r^	This study
pBBRCheYK^110R^	pBBRCheY carrying K110R substitution	This study
pBBRCheYK^110Q^	pBBRCheY carrying K110Q substitution	This study
pK18mobsacB	Suicide vector for gene deletion; *lacZ mob sacB* Km^r^	84
pRK2013	Helper plasmid, ColE1 replicon; Tra^+^ Km^r^	88
pK18mobsacB-*Δ0414*	pK18mobsacb with 575-bp upstream fragment and 516-bp downstream fragment of AZC_0414, Km^r^	This study
Strains		
Azorhizobium caulinodans		
ORS571	Wild-type strain; Amp^r^ Nal^r^	37
Δ*cheY1*	ORS571 derivative; deletion of *cheY1* (AZC_0620), Amp^r^ Nal^r^ Gm^r^	40, 48
*ΔAZC_0414*	ORS571 derivative; deletion of AZC_0414	This study

### Characterization of histone deacetylase AZC_0414 in *A. caulinodans* ORS571.

**FIG 7:**
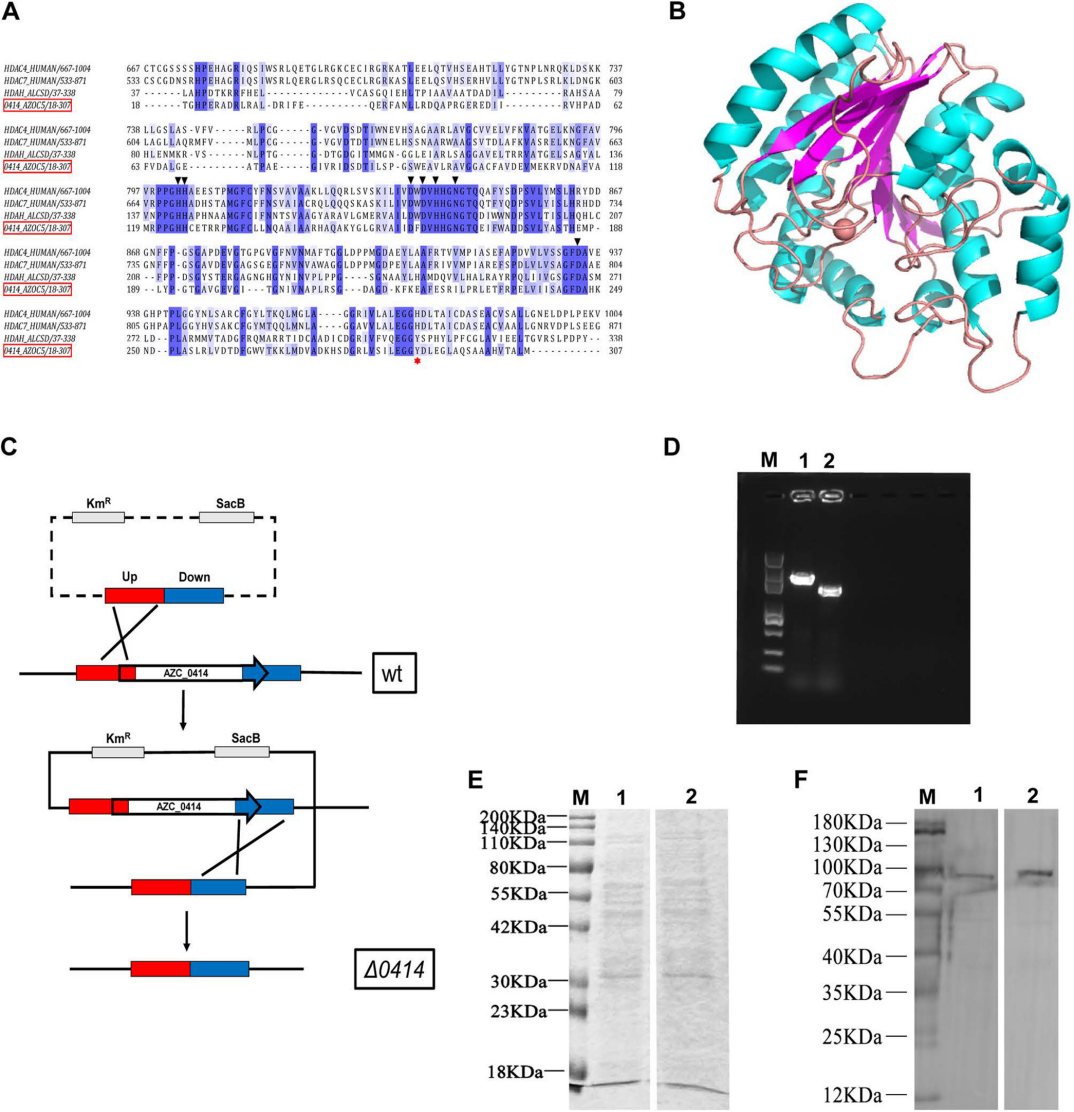
Characterization of Zn^2+^-dependent deacetylase AZC_0414 in A. caulinodans ORS571. (A) Sequence alignment of AZC_0414 with other Zn^2+^-dependent deacetylases. The backgrounds of conserved residues are marked with blue and light blue according to the degree of conservation. Black triangles represent conserved functional sites, and H976Y is represented by a red asterisk. (B) Predicted 3-D structure of deacetylase encoded by AZC_0414. The histone deacetylase superfamily protein 5ji5.1.A from B. phymatum (sequence identity = 45.57%) was identified as a template. The red sphere shows the zinc ion interacted with it. (C) Map of the *AZC_0414* markerless gene deletion. The upstream and downstream regions of the AZC_0414 gene are marked in red and blue. Km^r^, kanamycin resistance marker; SacB, sucrose counter-selectable marker. (D) PCR verification of the AZC_0414 gene knockout strain. M, DNA marker; lane 1, A. caulinodans ORS571; lane 2, A. caulinodans ORS571 *ΔAZC_0414*. (E) SDS-PAGE was used to analyze the protein expression of A. caulinodans ORS571 wild-type (lane 1) and AZC_0414 knockout (lane 2) strains. (F) Immunoblotting with anti-acetyl lysine antibody was used to analyze the lysine acetylation abundance of A. caulinodans ORS571 wild-type (lane 1) and AZC_0414 knockout (lane 2) strains.

Numerous acetylated protein substrates involved in various physiological processes were identified in the acetylome data described above. To further explore the regulatory mechanism of acetylation in A. caulinodans ORS571, a protein deacetylase-encoding gene, *AZC_0414*, was analyzed in detail. The sequence alignment showed that AZC_0414 was highly conserved with human HDAC4, HDAC7, and Bordetella/Alcaligenes HDAH (72–74) and had conserved active sites and zinc ion binding sites (Fig. 7A). Interestingly, human HDAC4 was an inefficient enzyme, but the catalytic activity of the H976Y mutant was increased 1,000 times (75). In A. caulinodans ORS571, the position corresponding to the H976 residue of human HDAC4 (marked with a red asterisk in Fig. 7A) was tyrosine (Y290 in AZC_0414). Therefore, we speculate that AZC_0414 may use acetylated lysine as a substrate with higher efficiency. The three-dimensional (3-D) structure of AZC_0414 was subsequently predicted. Homology modeling was performed using SWISS-MODEL with the template of histone deacetylase superfamily protein 5ji5.1.A from Burkholderia phymatum (sequence identity = 45.57%) (Fig. 7B). To investigate the physiological significance of AZC_0414, the knockout mutant strain was constructed (a concept map of gene knockout and PCR verification are shown in Fig. 7C and D). The relative protein abundances in the wild-type and *ΔAZC_0414* strains were determined using sodium dodecyl sulfate-polyacrylamide gel electrophoresis (SDS-PAGE), and the immunoblotting assay was performed using anti-acetylated lysine antibodies. As shown by the results in Fig. 7E, there was no significant difference in the expression of multiple proteins between the wild type and the *ΔAZC_0414* mutant. In the experiment whose results are shown in Fig. 7F, acetylated proteins were enriched in both the wild type and the *ΔAZC_0414* mutant using anti-acetylated lysine antibody, but the acetylation level of substrates in the *ΔAZC_0414* mutant was higher than that in the wild type. This indicates that AZC_0414 has deacetylase activity in A. caulinodans ORS571 and plays a role in protein deacetylation.

### Deletion of AZC_0414 affects the chemotactic motility of *A. caulinodans* ORS571.

**FIG 8:**
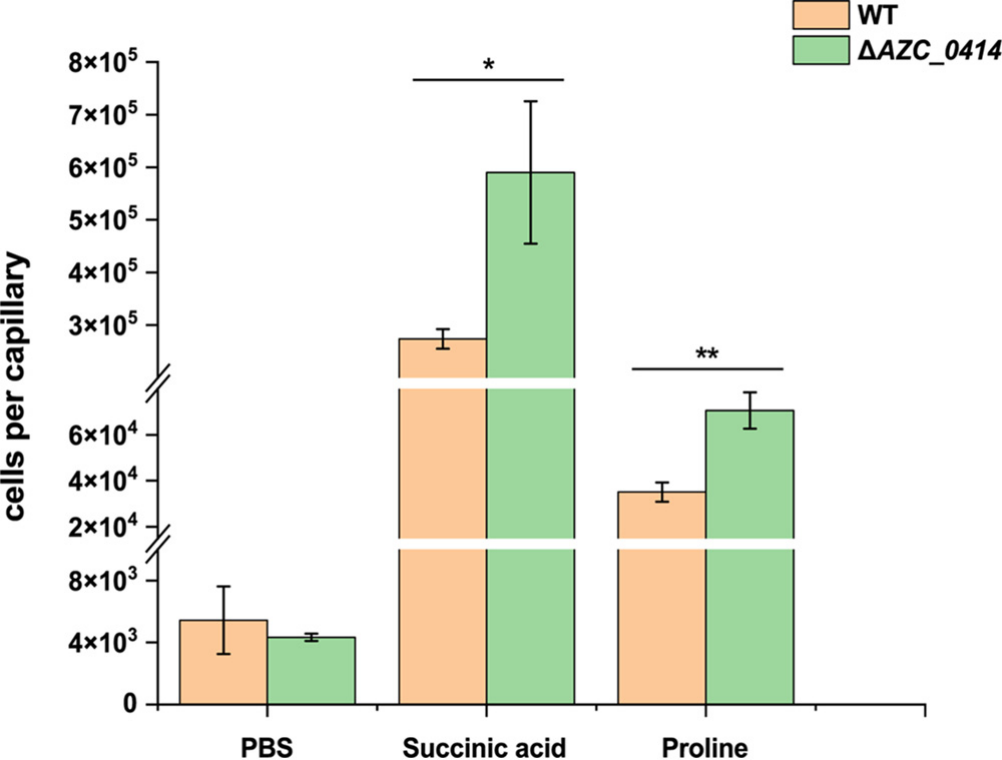
Chemotaxis of strain ORS571 and the *ΔAZC_0414* mutant. PBS was used as a control, while succinate and proline were used as chemoattractants. The chemotaxis of the wild type (WT) and the mutant was quantified using the capillary assay. Data are shown as the mean values and standard deviations from three independent experiments, and asterisks represent significant differences (*, *P* < 0.05; **, *P* < 0.01).

Previous studies have shown that the sirtuin CobB, as a global deacetylase, is involved in the regulation of various physiological processes, such as chemotaxis (33), stress response (1), and carbon source utilization (1, 15), but less research has been done on Zn^2+^-dependent deacetylases in bacteria. After biochemical analysis and characterization of the protein encoded by AZC_0414, the physiological function of the *ΔAZC_0414* mutant was investigated. As shown by the results in Fig. 8, deletion of AZC_0414 changed the chemotactic behavior in ORS571. Compared with the wild type, the *ΔAZC_0414* mutant showed significant increases in chemotactic motility toward succinic acid and proline. The wild type and the mutant showed no significant difference in chemotactic motility toward phosphate-buffered saline (PBS) (control check). It was reported that the deletion of CobB reduced the chemotactic ability of E. coli and further revealed that CobB regulated E. coli chemotaxis by deacetylating CheY (33). Therefore, we speculate that AZC_0414 may deacetylate proteins in the chemotaxis pathway or methyl-accepting chemotaxis protein (MCP) to regulate the chemotaxis of ORS571.

### Conclusions.

In this study, we provide the first extensive data on lysine acetylation in the rhizobium A. caulinodans strain ORS571. We identified a total of 2,302 acetylation sites from 982 proteins in A. caulinodans ORS571. Further functional studies revealed that reversible lysine acetylation of proteins was involved in numerous physiological processes, such as carbon metabolism, translation, chemotaxis, symbiotic nitrogen fixation, and quorum sensing. Conserved motifs of acetylated proteins were analyzed, and acetylated protein interaction networks were constructed. We further confirmed that the chemotaxis core protein CheY1 was regulated by acetylation in A. caulinodans ORS571. Significantly, the characterization of a new deacetylase, AZC_0414, showed the different chemotaxis behavior. Taken together, our results provide new insights into posttranslational modifications of proteins in rhizobia and provide an important basis for understanding the regulatory mechanisms of lysine acetylation in A. caulinodans ORS571.

## MATERIALS AND METHODS

### Strains and culture conditions.

A. caulinodans ORS571 wild-type strain was grown at 37°C in L3 minimal medium (20 mL/L 50% sodium lactate, 10 mL/L 1 M K-P_i_ buffer, 1 mL/L 1,000× Mg-Na-Mo mixture, 10 mL/L 1 M NH_4_CL, and water to a volume of 1 L, pH 7.0 [45]; sterilize, cool to about 50°C, and add trace elements CaCl_2_ and FeCl_3_ and vitamin mixture). The cells were cultured for about 26 h (until the stationary growth phase, optical density at 600 nm [OD_600_] of 3.0) in a well-aerated and free-living state, and then the protein samples were prepared. The cell density of the cultures was monitored by measuring the OD_600_.

### Protein extraction and digestion.

**(i) Protein extraction.** UA buffer (8 M urea, 100 mM Tris-HCl, pH 8.5) was used for sample lysis and protein extraction. Free-living bacteria were collected under good aeration, and the concentration of ammonia in the medium was 10 mM. The amount of protein was quantified with the Bradford protein assay kit.

**(ii) SDS-PAGE.** An amount of 20 μg of protein for each sample was mixed with 5× loading buffer and boiled for 5 min. The proteins were separated on 12.5% SDS-PAGE gels (constant current at 14 mA for 90 min). Protein bands were visualized by Coomassie blue R-250 staining.

**(iii) In-solution digestion.** dl-Dithiothreitol (DTT) with a final concentration of 10 mM was added to each sample, mixed at 600 rpm for 1.5 h (37°C), and then cooled to room temperature. Iodoacetamide (IAA) with a final concentration of 50 mM was added, and the mixture incubated in the dark for 30 min. The concentrate of UA buffer was diluted to 2 M with 4 times the volume of 50 mM Tris-HCl (pH 8.0). Trypsin was added to the samples at a trypsin/protein (wt/wt) ratio of 1:50 and incubated at 37°C for 15 to 18 h (overnight). After overnight digestion, trifluoroacetic acid (TFA) with a final concentration of 0.1% was added and the pH adjusted to ≤3 with 10% TFA. The digest peptides of each sample were desalted on C18 cartridges (Empore SPE C18 cartridges, bed inner diameter of 7 mm, 3-mL volume; Sigma) and lyophilized for further use.

### Acetylated-peptide enrichment.

The samples were reconstituted in 1.4 mL of precooled immunoaffinity purification (IAP) buffer (PTMScan IAP buffer; Cell Signaling Technology), pretreated anti-acetylated lysine (Ac-K) antibody beads (PTMScan acetyl-lysine motif [Ac-K] kit; Cell Signaling Technology) were added, the mixture was incubated at 4°C for 1.5 h and centrifuged at 2,000 × *g* for 30 s, and the supernatant discarded. Anti-Ac-K antibody beads were washed with 1 mL precooled IAP buffer 3 times and then washed with precooled water 3 times. An amount of 40 μL 0.15% TFA was added to the washed beads and incubated for 10 min at room temperature, and then 0.15% TFA was added again, the mixture centrifuged at 2,000 × *g* for 30 s, and the supernatant desalted by C18 STAGE (stop and go extraction) tips.

### LC-MS/MS analysis.

Liquid chromatography-tandem mass spectrometry (LC-MS/MS) analysis was performed on a timsTOF pro mass spectrometer (Bruker) that was coupled to a nanoElute system (Bruker Daltonics) for 60 min. The peptides were loaded on a C18 reversed-phase analytical column (homemade, 25 cm long, 75-μm inner diameter, 1.9-μm particle size, C18) in buffer A (0.1% formic acid) and separated with a linear gradient of buffer B (84% acetonitrile and 0.1% formic acid) at a flow rate of 300 mL/min. The mass spectrometer was operated in positive ion mode. The mass spectrometer collected ion mobility MS spectra over a mass range of *m/z* 100 to 1,700 and 1/K_0_ of 0.6 to 1.6, and then 10 cycles of parallel accumulation-serial fragmentation (PASEF) MS/MS were performed with a target intensity of 1.5 K and a threshold of 2,500. The active exclusion was enabled with a release time of 0.4 min.

### Identification and quantitation of modified proteins.

The MS raw data for each sample were combined and searched using the MaxQuant software (version 1.5.3.17) for identification and quantitation analysis. Related parameters and instructions are as follows. Two missed cleavages were allowed for trypsin, the ion mass tolerance was set to 20 ppm, carbamidomethylation (Cys) was set as a fixed modification, and oxidation (M) and acetylation (K) were considered variable modifications. The false discovery rate (FDR) thresholds for proteins, peptides, and modification sites were specified at 1%. The time window (match between runs) was set to 2 min. Then, 4,711 sequences in the database (Uniprot-taxonomy_Azorhizobium_caulinodans_4711_20210429.fasta) were searched using the above-described parameters.

### Bioinformatic analysis.

All proteins involved in bioinformatics analysis in the acetylome data had acetylation sites identified with high confidence (FDR of <1% and acetyl probabilities of >0.75). Subcellular localization analysis of acetylated proteins was performed by using the subcellular structure prediction software CELLO (76). The annotation of the acetylated proteins is divided into three parts: Gene Ontology (GO), structural domain, and Kyoto Encyclopedia of Genes and Genomes (KEGG) (77). The protein sequences of the selected acetylated proteins were searched locally using the NCBI BLAST+ client software (NCBI-blast-2.2.28+-win32.exe) and InterProScan to find homologue sequences, and then GO terms were mapped and sequences were annotated using the software program Blast2GO (78). The GO annotation results were plotted by using R scripts. The KEGG database (http://www.genome.jp/kegg/) was used to retrieve the acetylated proteins for orthologs identified by the Basic Local Alignment Search Tool (BLAST), and the annotation results were subsequently mapped to pathways in KEGG. The domain annotation of acetylated proteins was performed by using InterProScan software. InterProScan integrates some of the most widely used databases; the Pfam database (https://pfam.xfam.org/) was used to characterize acetylated protein domains in this study (79).

The MEME software (80) was used to analyze the conserved motifs around the acetylation sites. Relative to motif-x, improved statistical confidence estimates and more accurate calculation of motif scores were provided by this software. The modification site and six amino acids upstream/downstream from the modification site (13 amino acid sites in total) were extracted and used to predict motifs in this study (parameters were as follows: occurrences = 50, significance = 1e−06, background = UniProt-taxonomy_Azorhizobium_caulinodans_4711_20210429). In addition, evolutionary conservation analysis of acetylated proteins was performed by BLASTP. The acetylated protein sequences of A. caulinodans strain ORS571 were compared with the sequences of E. coli and yeast, and the protein homologues were screened and displayed.

Acetylated proteins were mapped to the STRING database (http://string-db.org/) for analysis of protein-protein interactions (PPI). All interactions with high confidence (confidence score of ≥0.7) in the STRING database were screened, and the results were downloaded and imported into the Cytoscape software (http://www.cytoscape.org/, version 3.9.1) for visualization. The Cytoscape plugin MCODE (Molecular Complex Detection) was used to find clusters in the network.

### Chemotactic motility assay.

To construct the CheY1 mutant, the CheY1 target fragment or its acetylation site mutant was amplified and cloned into the broad-host-range plasmid pBBRMCS-2. To avoid the interference of the *cheY1* gene on the chromosome, the resulting plasmids were transferred into the *ΔcheY1* strain. Previous studies have shown that the *ΔcheY1* strain in the A. caulinodans strain ORS571 background completely loses motility (40). The phenotypes of the complemented strains with different CheY1 fragments are able to show the effect of the corresponding modification on the chemotaxis of ORS571.

The soft agar plate method used for the motility assay was modified based on the previous study (81). A. caulinodans ORS571 and the mutants grown to mid-log phase were washed with double-distilled water, and the cell density was adjusted to an OD_600_ of 0.6. Amounts of 5 μL of the bacterial suspensions were inoculated into the soft agar plates (0.3% agar) containing different carbon sources (TY and L3+N medium [45, 82]) and incubated at 37°C for 48 h before the swimming ring of the strains was measured.

### Western blotting with anti-acetyl lysine antibody.

A. caulinodans ORS571 and the mutants were grown at 37°C in L3 minimal medium with sodium lactate. Cells were harvested by centrifugation and lysed using radioimmunoprecipitation assay (RIPA) lysis buffer (product number G2002; Servicebio) supplemented with the protease inhibitor. The protein concentration was measured using the bicinchoninic acid (BCA) protein concentration assay kit (product number G2026; Servicebio). Extracted proteins were separated by 10% SDS-PAGE and transferred to a polyvinylidene difluoride (PVDF) membrane. The transferred membrane was placed in an incubation tank containing TBST (10 mM Tris, 150 mM NaCl, 0.1% Tween 20, pH 7.4 to 7.6) and blocked with skim milk powder for 30 min at room temperature. Rabbit anti-acetylated lysine polyclonal antibody (#9441; Cell Signaling Technology) was diluted 1:1,000 as the primary antibody, and the membrane was incubated overnight at 4°C in a shaker. Horseradish peroxidase (HRP)-conjugated goat anti-rabbit antibody (GB23303; Servicebio) was used as the secondary antibody, diluted at a ratio of 1:5,000, and the membrane was washed with TBST and incubated with the diluted secondary antibody for 30 min at room temperature in a shaker. TBST-washed membranes were developed using enhanced chemiluminescence (ECL), followed by gel image analysis.

### Construction of the *ΔAZC_0414* mutants in *A. caulinodans* ORS571.

The knockout mutant of A. caulinodans ORS571 was generated by triparental mating as previously described, with some modifications (83). The sucrose-sensitive plasmid pK18mobsacB (84) was used for the homologous recombination-mediated deletion of the *AZC_0414* (AZC_RS02110) gene, which encodes a histone deacetylase in A. caulinodans ORS571. About 500 bp immediately upstream and downstream from the deleted codons were amplified and combined by using 2× Phanta max master mix (dye plus) (Vazyme Biotech Co., Ltd.). The PCR products were then cloned into pK18mobsacB. The plasmid was transformed into chemically competent Escherichia coli cells (TransGen Biotech), and the donor strain was obtained by screening with kanamycin. The plasmid containing the deleted fragment was introduced into A. caulinodans ORS571 cells under the mediation of the donor strain and the helper strain containing the plasmid PRK2013 (85). Mating of the three strains was performed on antibiotic-free TY solid medium, and the cultures incubated at 37°C for 48 h. The bacteria were collected, diluted, spread on the TY solid plates containing 100 μg/mL ampicillin and 100 μg/mL kanamycin (Solarbio), and cultured at 37°C for 3 days. Single colonies verified by PCR were inoculated into TY liquid medium without antibiotics and subcultured at 37°C for about 20 h. The cultures were then plated on kanamycin- and sucrose-containing TY solid plates, and those that could not grow on the kanamycin resistance plates but could grow on 10% sucrose plates were selected. The mutant strain was confirmed with PCR using the FastPure blood/cell/tissue/bacteria DNA isolation minikit (Vazyme Biotech Co., Ltd.) and DNA sequencing.

### Capillary quantification assay.

The quantitative assay for chemotaxis was designed on the basis of the modified capillary assay (86). The wild-type and mutant strains were grown at 37°C in L3 minimal medium. The cells were cultured for about 20 h and then washed by chemotaxis buffer, and the cell density was adjusted to an OD_600_ of 0.1. Amounts of 100 μL of bacterial suspension and chemoattractant (succinic acid or proline) were loaded to 200-μL tips and 1-mL syringes, respectively. The tips and syringes were connected together head-to-head for 30 min. Cells swimming into the syringes were measured using plate counting.

### Statistical analysis.

Data among different treatments were analyzed by using IBM SPSS Statistics 21. Fisher’s analysis of variance (ANOVA) and the least significant difference (LSD) *post hoc* test (*P* < 0.05 or *P* < 0.01) were used to determine significant differences between treatments.

### Data availability.

The mass spectrometry proteomics data have been deposited to the ProteomeXchange Consortium (http://proteomecentral.proteomexchange.org) via the iProX partner repository with the data set identifier PXD031018.
