# Supplementary material for: Systematic Analysis of Lysine Acetylation Reveals Diverse Functions in Azorhizobium caulinodans Strain ORS571
Source: Microbiol Spectr. 2022 Dec 8;11(1):e03539-22. doi: 10.1128/spectrum.03539-22 (PMC9927263; doi:10.1128/spectrum.03539-22)
Supplement: Supplemental file 2 — Fig. S1 and S2. Download spectrum.03539-22-s0002.pdf, PDF file, 0.4 MB [file spectrum.03539-22-s0002.pdf]

Supporting Information

Systematic Analysis of Lysine Acetylation Reveals Diverse Functions in *Azorhizobium caulinodans* ORS571

Yanan Liu[1,3], Xiaolin Liu[1], Xiaoyan Dong[1], Zhiqiu Yin[2], Zhihong Xie[2*], Yongming Luo[4]

1. CAS Key Laboratory of Coastal Environmental Processes and Ecological Remediation, Yantai Institute of Coastal Zone Research, Chinese Academy of Sciences, Yantai 264003, China

2. National Engineering Research Center for Efficient Utilization of Soil and Fertilizer Resources, College of Resources and Environment of Shandong Agricultural University, Taian 271000, China

3. University of Chinese Academy of Sciences, Beijing, China

4. CAS Key Laboratory of Soil Environment and Pollution Remediation, Institute of Soil Science, Chinese Academy of Sciences, Nanjing 210008, China

*Corresponding Author Email： zhihongxie211@163.com

Table of Contents

Table S1 Overview of identified acetylated proteins and peptides.

Table S2 Gene ontology cluster analysis of acetylated proteins.

Table S3 Domain cluster analysis of acetylated proteins.

Table S4 Metabolic pathways analysis of acetylated proteins.

Table S5 Acetylated proteins with special ecological significance in *A. caulinodans* ORS571

Table S6 Motif type of acetylated peptides.

Table S7 Ortholog acetylated proteins of *A. caulinodans* ORS571 with *E.coli* and yeast.

Table S8 Analysis of acetylated proteins interaction networks.

Supporting Figures

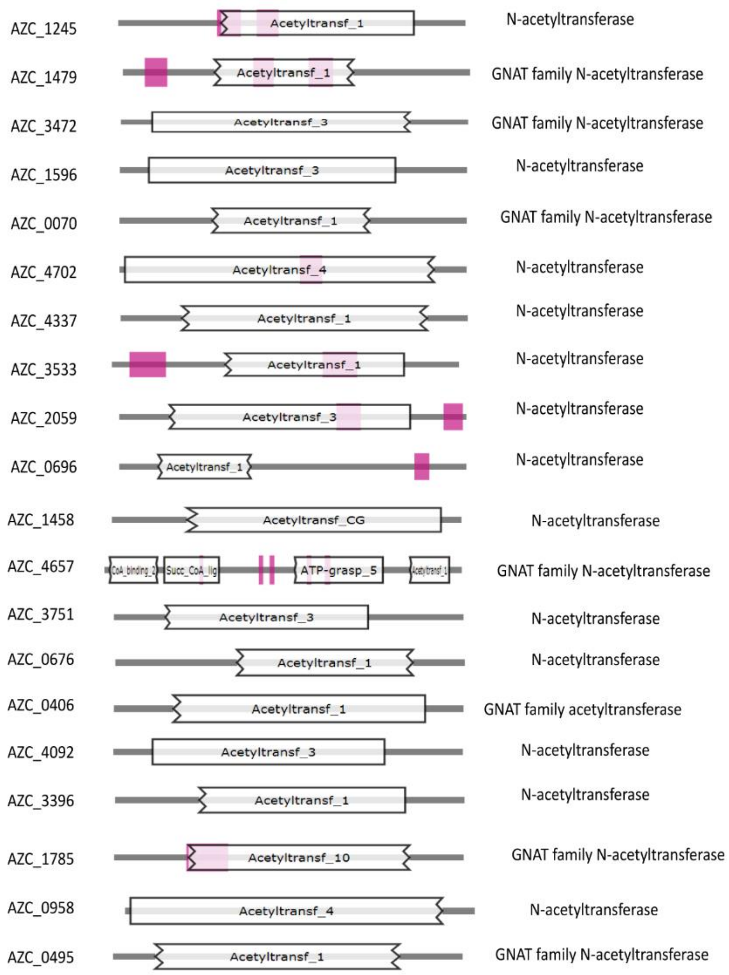

Figure S1 Putative lysine acetyltransferases in the genome of *A. caulinodans* ORS571. Domain structures were predicted by using the SMART program.

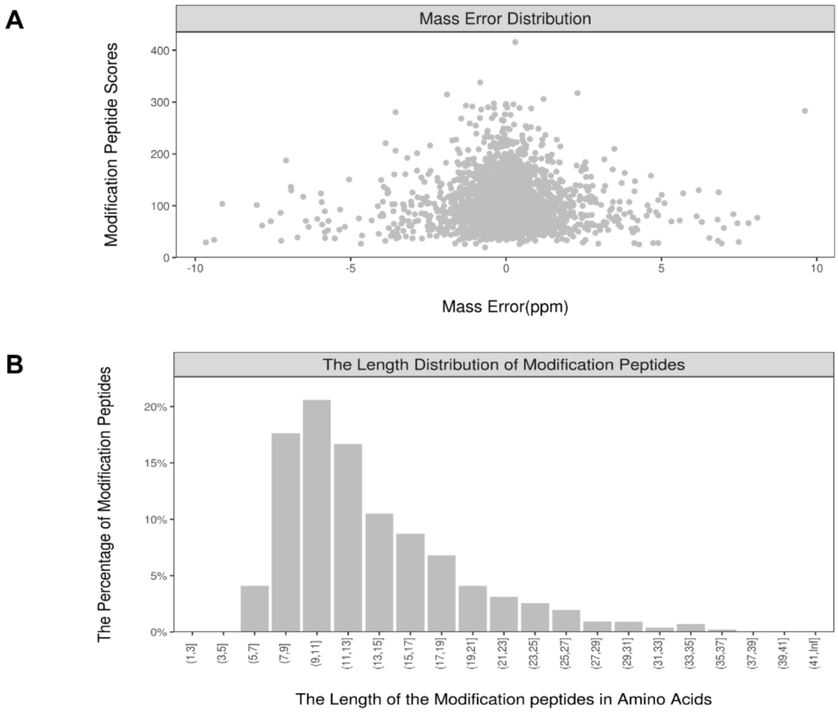

Figure S2 Quality control of MS spectrum data. (A) The mass deviations of all identified acetyl-peptides were mainly within 10 ppm, indicating that the quality accuracy of the MS data is reliable. (B) Most of the peptides were distributed between 7 and 20 amino acids in length, which is consistent with the digestive properties of trypsin.
